# Effects of blood loss on organ attenuation on postmortem CT and organ weight at autopsy

**DOI:** 10.1007/s00414-021-02731-8

**Published:** 2021-11-24

**Authors:** Jakob Heimer, Vasiliki Chatzaraki, Wolf Schweitzer, Michael J. Thali, Thomas D. Ruder

**Affiliations:** 1 Institute of Forensic Medicine, Department of Forensic Medicine and Imaging, University of Zurich, Winterthurerstrasse 190/52, 8057 Zurich, Switzerland; 2 Department of Diagnostic, Interventional and Pediatric Radiology, Inselspital, Bern University Hospital, University of Bern, Freiburgstrasse 18, 3010 , Bern, Switzerland

**Keywords:** Postmortem computed tomography, Autopsy, Exsanguination, Organ attenuation, Organ weight

## Abstract

**Background:**

Cases of external hemorrhage are difficult to recognize on postmortem computed tomography (PMCT).

**Purpose:**

To investigate the effects of blood loss on CT attenuation of the spleen, liver, kidneys, and lungs on PMCT and to assess the relationship between blood loss and organ weight.

**Methods:**

A total of 125 cases with blood loss were sex- and age-matched to 125 control cases without blood loss. Individual organ attenuation was measured on transverse CT images. Organ weights of the liver, spleen, kidneys, and lung were extracted from the autopsy protocols.

**Results:**

Organ weight was significantly lower in cases with blood loss (lung 30%, spleen 28%, kidneys 14%, liver 18%) than in controls. CT attenuation of the lungs was significantly lower (30%) in cases with blood loss than in controls. CT attenuation of the spleen and kidneys did not significantly differ between cases and controls. CT attenuation of the liver was significantly higher (25%) in cases with blood loss than in controls.

**Conclusion:**

Blood loss decreases organ weight and CT attenuation of the lungs but appears to have no significant effect on CT attenuation of the spleen and kidneys. The increased liver attenuation in cases with blood loss compared to controls was an unexpected finding and remains challenging to explain. One probable interpretation refers to different levels of hepatic glycogen; however, further work is warranted to substantiate this hypothesis.

## Introduction

Hemorrhage is a critical component of accidental and inflicted trauma but may also occur in cases of vascular ruptures due to malformation, inflammation, or neoplasia. The human body contains approximately 3.5–6.5 L of blood [1, 2], depending on sex and body mass. A loss of more than 40% of the total blood volume may lead to profound hypovolemic shock and death [3]. Significant hemorrhage and exsanguination are associated with typical findings at external inspection and autopsy. The loss of blood translates to a decreased outer lividity [4], and inner organs present with a distinctive pallor, particularly those with voluminous capillary systems, such as the liver, spleen, and kidneys [4]. It is not entirely clear if hemorrhagic hypovolemia affects organ weight as measured at autopsy. While comprehensible in theory, a decrease in organ weight due to blood loss has only been demonstrated by Boyd et al. [5] and Moar et al. [6], while Myers and Segal, as well as Hadley et al., did not consider hemorrhagic hypovolemia as a cause of observed differences in organ weight [7, 8].

Although hemorrhage and exsanguination may already be suspected after the external post-mortem examination, blood loss is surprisingly difficult to recognize on postmortem computed tomography (PMCT). Particularly external hemorrhage is often elusive on PMCT. To date, only a few radiologic signs of substantial blood loss have been reported. Aghayev et al. observed a collapse of central vessels in cases of fatal hemorrhage [9]. This finding was later confirmed by Sogawa et al. [10], who also introduced and computed a vessel flattening index. Schober et al. investigated the attenuation of lungs on PMCT with respect to the cause of death and observed decreased Hounsfield unit (HU) values both in cases of fatal hemorrhage and hypothermia [11].

The idea of measuring CT attenuation profiles of specific organs on PMCT in an attempt to link different HU values to different causes of death is very alluring and has also been tested for the liver and the spleen with moderate success [12, 13]. The main limitation of previous work on this topic was that most causes of death do not alter the composition of these organs. This is crucial because the attenuation of an absorber (such as an organ) on CT is determined by the composition of the absorber as well as by CT scan parameters (especially CT beam energy) [14].

Based on these fundamental principles of CT physics [14] and Schober’s work [11] on lung attenuation, this study hypothesized that hemorrhage—i.e. the removal of blood from organs—would decrease mean attenuation values of parenchymatous organs.

This study aimed to assess the effect of hemorrhage (i.e., the removal of blood) on CT attenuation of the spleen, liver, and kidneys on PMCT. To validate the methodology, the—known—effect of blood loss on lung attenuation was also evaluated. The second aim of the study was to assess the relationship between blood loss and organ weight.

## Methodology

### Case and control case selection and matching

The institutional electronic case records were searched (period from 2015 to 2019) for cases with documented blood loss (either as the primary cause of death or in conjunction with lethal trauma) and for control cases with no blood loss. Each hemorrhage cases was sex- and age-matched (± 1 year) with a non-hemorrhage control case (referred to as cases and controls in the remaining manuscript). Individuals with successful on-scene resuscitation and delayed death after hospitalization were excluded. Attempted cardiopulmonary resuscitation (CPR) was noted. Cases were also excluded in the presence of any residues of contrast media from clinical CT examinations or postmortem angiography, visible lacerations, neoplastic infiltration, and structural pathology (e.g., cirrhosis, fibrosis) of any of the target organs, streak artifacts from metallic implants, severe intrahepatic gas, and any gas in the spleen or kidneys signaling putrefaction or injury. A total of 125 cases for each group was included in the study. For each case, the time of death, time of PMCT, age, sex, height, weight, and estimated postmortem interval in (PMI) were extracted from the electronic case records. The body mass index (BMI) was calculated from height and weight.

### Computed tomography

**Fig. 1:**
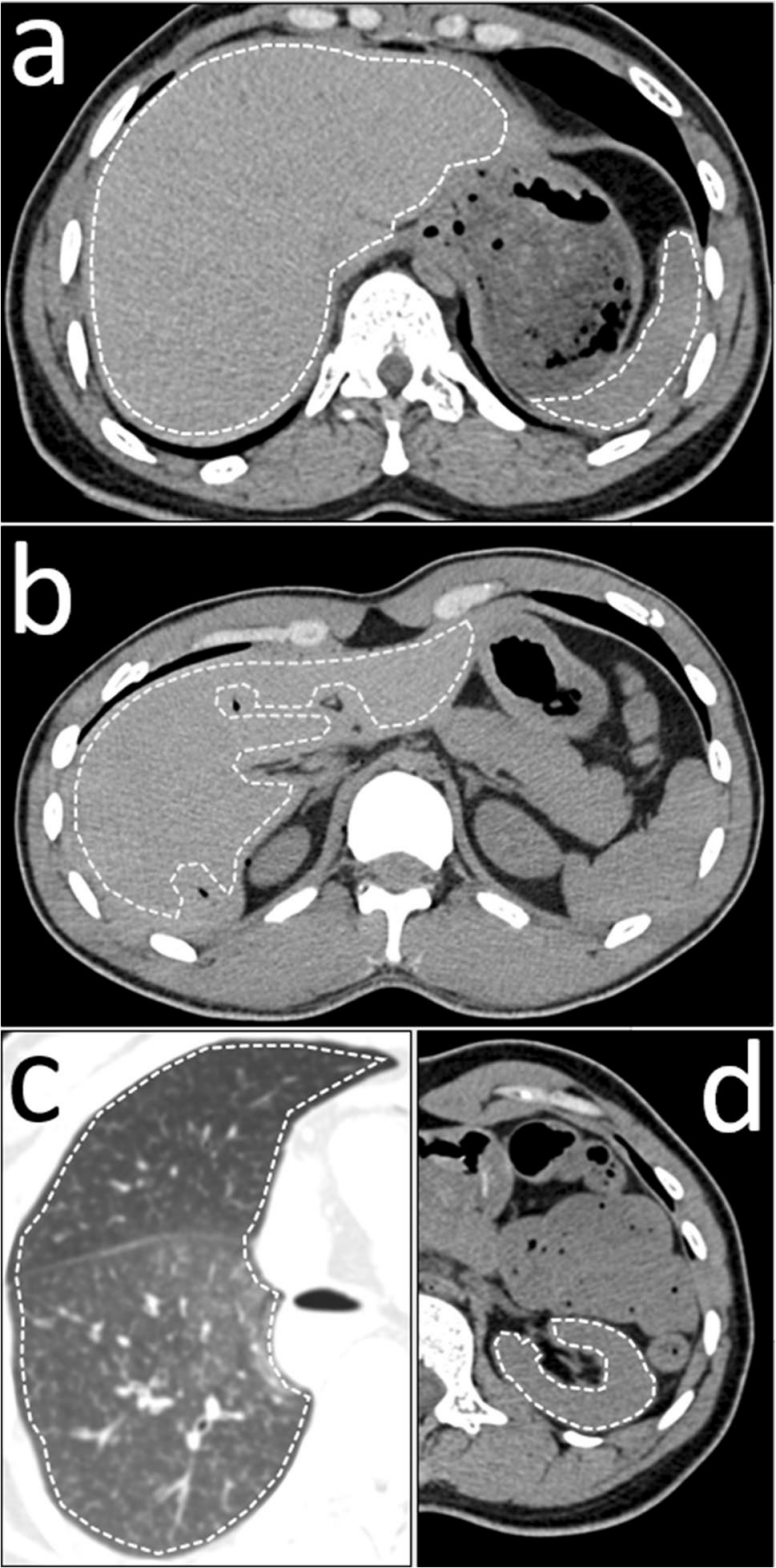
Measured regions of interest. The liver and spleen were measured on the level of the eleventh thoracic vertebra with a maximum transverse ROI (**a**). Intrahepatic gas and the teres hepatis ligament were excluded from the ROI (**b**). The lung was measured on the first transverse slice that did not show any part of the liver (**c**). The left kidney was measured on the level of the first lumbar vertebra (**d**)

Non-contrast whole-body PMCT was performed using a 128-slice scanner (Somatom Definition Flash, Siemens Medical Solutions, Forchheim, Germany). The reconstruction parameters were 120 kV, 1-mm slice thickness, 0.6-mm increment, and a 445 × 445 mm field of view. The scanner was regularly calibrated to a water standard. Reconstructions were calculated in a soft and a hard kernel. For each case, regions of interest (ROI) were manually placed on predefined transverse slices of the right lung, liver, left kidney, and spleen (Fig. 1). The effect of intraparenchymal blood sedimentation as a confounding factor for ROI measurements, as reported by Jackowski et al., was prevented by measuring the entire transverse organ area with a non-circular, polygonal ROI [15]. Although circular ROI are more frequently used for HU measurements than non-circular polygonal ROI in the literature, earlier work by Ruder et al. has shown that the shape of the ROI does not have a significant influence on HU value measurements [16]. The liver and spleen were measured at the level of the 11th thoracic vertebra with a maximum transverse ROI. The teres hepatis ligament and marginal hepatic gas accumulations were not included in the ROI. The left kidney was measured at the level of the first lumbar vertebra. The lung was measured on the last transverse slice cranial to the dome of the diaphragm. In the case of a right-sided pneumo- or hemothorax, the left lung was measured. For each ROI, the mean HU unit value was recorded. To test the inter-rater reliability of the methodology, a second rater repeated the polygonal ROI measurements for all livers with the same approach. For the resulting values, a two-way intraclass correlation coefficient (ICC) was 0.992 (*F* = 257, *p* < 0.001). The mean HU value of both liver measurements is the number reported in this study.

### Autopsy

A board-certified forensic pathologist and a resident or two certified forensic pathologists conducted the autopsy according to the institutional standard. Height, body weight, and organ weights were noted. Both kidneys were weighed together. As per autopsy protocol, hepatic steatosis was classified visually by a forensic pathologist as 0 (no steatosis), 1 (mild steatosis), 2 (moderate steatosis), or 3 (severe steatosis).

### Statistical analysis

The matching of cases and controls was assessed with standardized mean differences (SMD). Variables with an SMD of < 0.1 were considered balanced across cases and controls. The mean values of continuous variables were compared with *t*-tests, and the proportions of categorical variables were compared by chi-square tests. Multiple regression analysis was used to determine the associations of predictors and the CT attenuation of the liver and the lungs (dependent variable or “response”), and to adjust for confounders [17]. Predictors in these models were the group membership (cases or controls), hepatic steatosis, sex, age at death, liver and lung weight, BMI, and PMI, and attempted CPR. To enable a comparison of the relative strength of the association of the predictors and the attenuations of the lungs and the liver, the partial standardized coefficients (beta coefficients) were computed and plotted [18]. In the subgroup analysis of liver attenuation, the comparisons between the mean attenuations were achieved by an ANOVA followed by a Tukey-HSD test. A *p* value < 0.05 was considered significant. All analyses and plots were computed with R [19].

## Results

### Study population

**Fig. 2:**
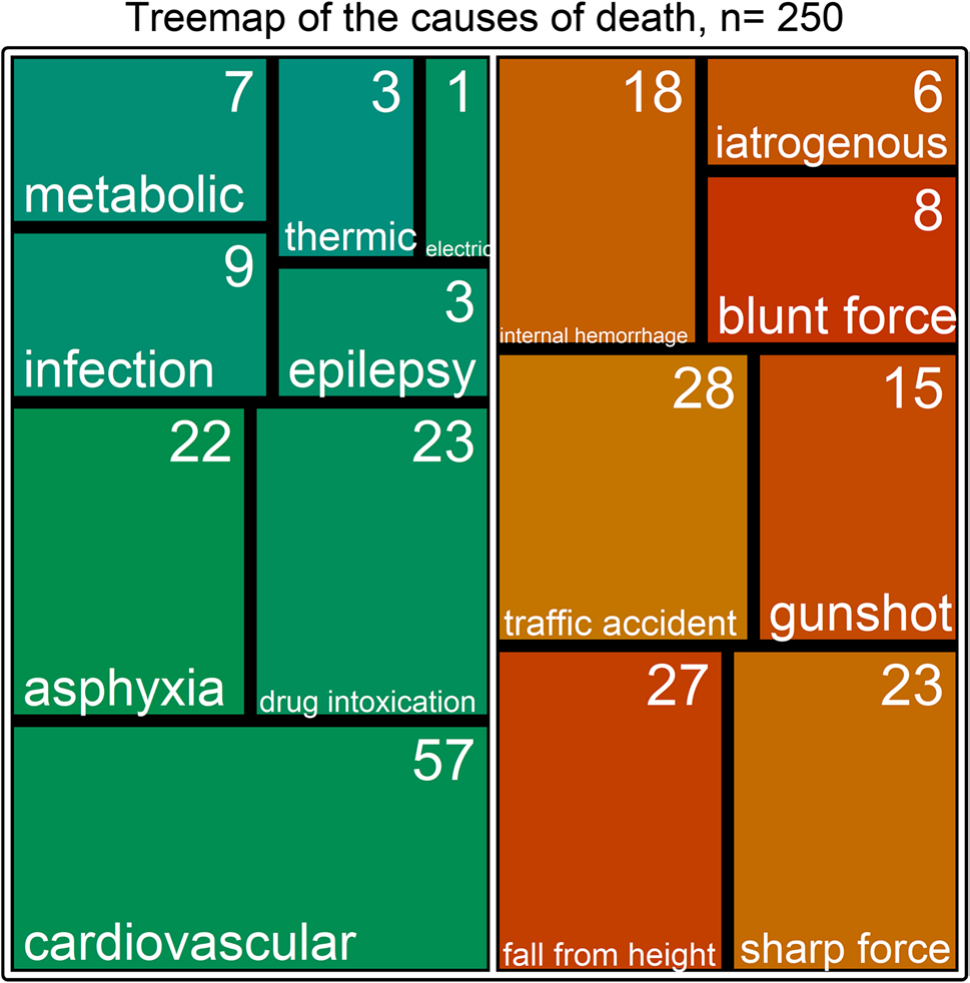
Treemap of the causes of death, *n* = 250; red/orange, cases; green/blue, controls; white text and numbers, classification of cause of death and numbers of cases

A complete overview of the generated data is shown in Table 1. With respect to the SMD, the matching variables (sex and age, SMD ~ 0, *p* ~ 1), and height (SMD = 0.06, *p* = 0.65) were balanced. Weight (SMD = 0.13, *p* = 0.323) and BMI (SMD = 0.1, *p* = 0.42) showed minor, non-significant imbalances. The number of attempted CPR (SMD = 0.37, *p* = 0.007) was significantly higher in the control group than in the case group. Hepatic steatosis was slightly more prevalent in the case group (44.0%) than in the control group (35.2%; SMD = 0.18, *p* = 0.18). The causes of death for cases and controls are presented in Fig. 2.

**Table 1:** Collected data with respect to the case and control groups

	Control Group	Case Group	*p*	SMD
n	125	125		
Sex = Female (%)	38 (30.4)	38 (30.4)	1.000	< 0.001
Age (mean (SD))	54.40 (18.73)	54.16 (18.93)	0.919	0.013
Weight (mean (SD))	76.24 (19.36)	74.03 (15.74)	0.323	0.125
Height (mean (SD))	173.46 (9.19)	172.92 (9.56)	0.652	0.057
BMI (mean (SD))	25.18 (5.47)	24.67 (4.55)	0.424	0.101
PMI (mean (SD))	30.68 (31.71)	26.56 (27.70)	0.274	0.139
Liver fat (%)			0.619	0.169
0	80 (64.0)	70 (56.0)		
1	19 (15.2)	23 (18.4)		
2	22 (17.6)	26 (20.8)		
3	4 (3.2)	6 (4.8)		
REA = 1 (%)	58 (46.4)	36 (28.8)	0.006	0.370

### Organ attenuation and weights

Univariate results for the organ attenuations and weights are displayed in Table 2. The lung attenuation of the cases was significantly lower (− 153.7, 30%, *p* < 0.001) than that of the controls, while the liver attenuation was significantly higher (12.2 HU, 25%, *p* < 0.001) than that in controls. The spleen and kidney attenuations did not show significant differences among cases and controls. The weights of all organs in the case group were significantly lower than the organ weights in the control group (*p* < 0.001) with a mean weight difference between the cases and controls of 30.4% for the lung, 17.7% for the liver, 27.7% for the spleen, and 14.0% for the kidneys.

**Table 2:** Results of the univariate comparison of mean organ attenuation and weight

	Control Group	Case Group	p
*n*	125	125	
Liver attenuation (mean (SD))	49.25 (10.84)	61.43 (13.35)	< 0.001
Lung attenuation (mean (SD))	− 501.82 (157.30)	− 655.58 (129.69)	< 0.001
Spleen attenuation (mean (SD))	50.10 (6.33)	50.84 (6.20)	0.352
Kidney attenuation (mean (SD))	41.57 (5.83)	40.90 (5.65)	0.362
Liver weight (mean (SD))	1810.16 (482.60)	1489.18 (347.79)	< 0.001
Lung weight (mean (SD))	1529.68 (435.06)	1064.88 (330.22)	< 0.001
Spleen weight (mean (SD))	194.22 (100.40)	140.36 (58.73)	< 0.001
Kidney weight (mean (SD))	321.05 (85.29)	275.98 (65.61)	< 0.001

### Multiple linear regression analysis

**Fig. 3:**
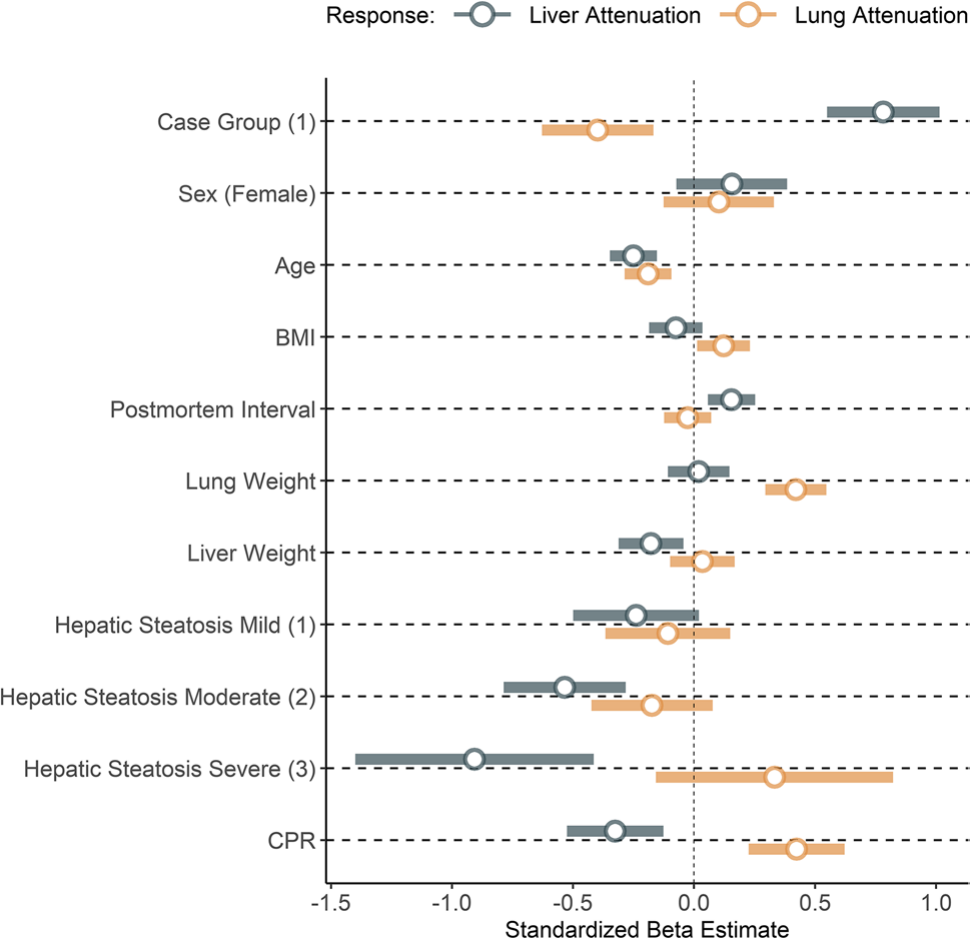
Overview of the beta coefficients of the regression models as a measure of relative strength of association of the predictors and the of the models for liver and lung attenuation. To obtain these beta-coefficients, the organ attenuations (responses) as well as continuous predictors were mean-centered and scaled by 1 standard deviation (standardized)

The summaries of the multiple linear regression models (analyses) for liver and lung attenuation are presented in Table 3 and Fig. 3. The group membership to the case group was a highly significant (*p* < 0.001) predictor for lung (− 64.3 HU) and liver (+ 10.62 HU) attenuation. Moderate (− 7.26 HU, p < 0.001) and severe hepatic steatosis (− 12.32 HU, *p* < 0.001) were significant predictors for liver attenuation. Attempted CPR, age, and BMI were significant predictors in both models, while female sex was a significant predictor for lung attenuation, and PMI was a significant predictor for liver attenuation. Lung weight was a significant predictor in the lung model with a positive coefficient (0.15, *p* < 0.001), while liver weight was a significant predictor in the liver model with a negative coefficient (− 0.01, *p* < 0.01).

**Table 3:** Outputs from the multiple linear regression models for lung and liver attenuation

	Liver attenuation	Lung attenuation
Case Group (1)	10.62 ***	-64.93 ***
Sex (Female)	2.12	16.75
Age	-0.18 ***	-1.65 ***
BMI	-0.2	3.97 *
PMI	0.07 **	-0.14
Lung Weight	0.00	0.15 ***
Liver Weight	-0.01 **	0.01
Hepatic Steatosis - Mild	-3.25	-17.67
Hepatic Steatosis - Moderate	-7.26 ***	-28.33
Hepatic Steatosis - Severe	-12.32 ***	54.29
Reanimation (1)	-4.42 **	69.19 ***
N	250	250
R2	0.49	0.50

^***^ *p* < 0.001; ** *p* < 0.01; * *p* < 0.05

For the liver model, the standardized coefficients (relative strength of association) of liver fat content (− 0.24, − 0.53, − 0.91) and the group membership case-group (0.78) followed by CPR (− 0.33) were the largest (Fig. 3). For the lung model, the standardized beta coefficients were largest for lung weight (0.42), attempted reanimation (0.42), and the case-group membership (− 0.4).

### Liver attenuation of subgroups

**Fig. 4:**
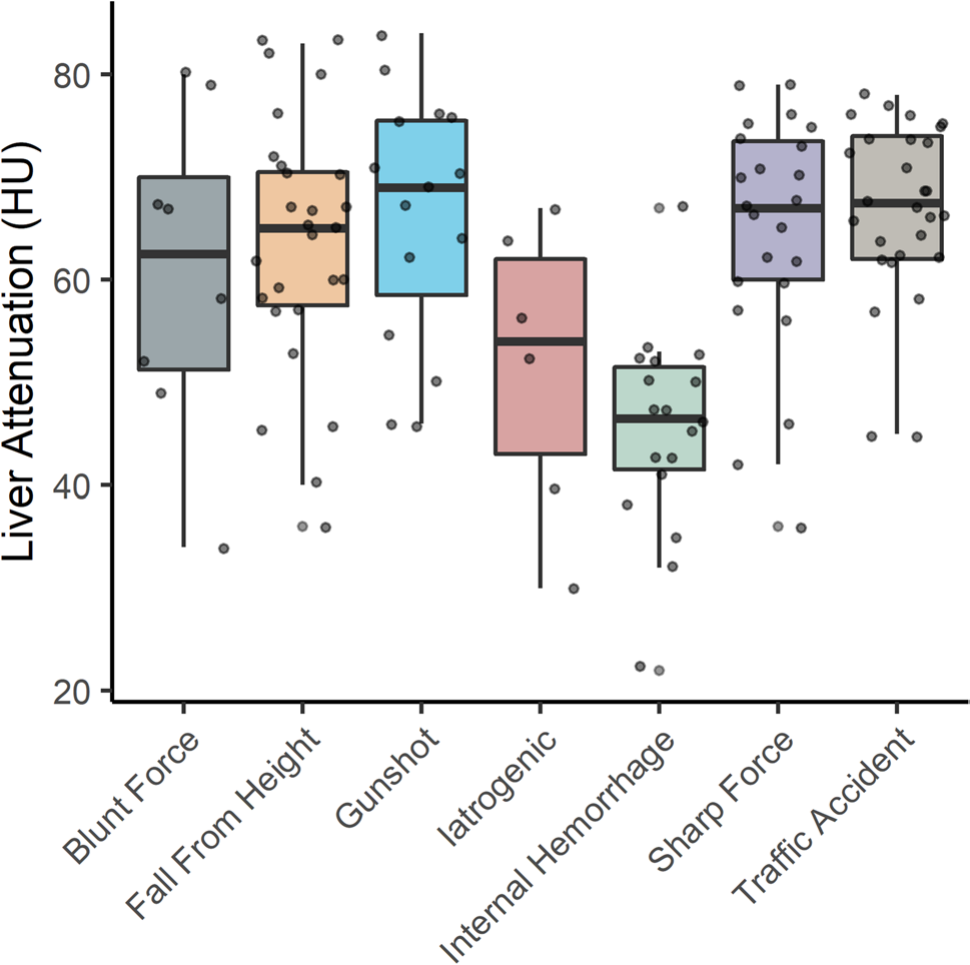
Boxplots of the liver attenuations in the case group stratified by the cause of death. Iatrogenic and internal hemorrhages present with lower average attenuation than all other causes of death

In Fig. 4, the boxplots of the liver attenuation with respect to the causes of death in the case group are shown. The subgroups “internal hemorrhage” and “iatrogenic” present with lower average attenuation than all other causes of death. The mean attenuation value for internal hemorrhage is significantly lower (*p* < 0.03 for all comparisons) for all other causes of death but for iatrogenic.

## Discussion

This postmortem case–control study investigated the effect of blood loss on CT attenuation values and the weight of the spleen, liver, kidneys and lungs on PMCT and found higher hepatic attenuation values and lower pulmonary attenuation values in cases with blood loss than in controls. No significant change in splenic and renal attenuation between cases and controls was observed. The weight of all organs was significantly lower in cases than in controls.

The difference in organ weight between the case group and control group strongly indicates that blood loss results in significant organ weight loss. This conclusion may appear trivial and intuitive; however, this issue has been discussed controversially in the existing literature [5–8]; our results support the findings of Boyd et al. [5] and Moar et al. [6].

As stated in the introduction, the attenuation of an organ is determined by the composition of the organ and by CT beam energy [14]. The effect of changing the composition of an organ was illustrated in the lungs: after the removal of blood (mean attenuation: 60 HU [20]), the relative contribution of intrapulmonary air (− 1000 HU, [14]) on lung attenuation increased sufficiently to decrease the mean attenuation value of the lungs. Our findings on lung attenuation are in agreement with those of Schober et al. [11]. This point is important because it underlines the validity of the methodology. However, decreased attenuation of the lung on PMCT is not specific for blood loss but also observed in hypothermia and obstructive asphyxia [11, 21, 22].

In contrast to the results on pulmonary attenuation, this study found no significant difference in renal and splenic attenuation between cases and controls. This finding suggests that the attenuation of blood does not significantly differ from the attenuation of renal and splenic parenchyma. The findings on splenic attenuation are indirectly corroborated by Chatzaraki et al., who reviewed splenic attenuation and size in correlation to various causes of death [13].

The high liver attenuation value in the case group after blood loss was an unexpected finding. Mean liver attenuation was approximately 12 HU higher in cases with blood loss (61 HU) than controls without blood loss (49 HU). This difference in means is reinforced in the multiple regression analysis. The binary predictor “group membership: Case Group” is highly significant in both regression models. It is negatively associated with lung attenuation and positively associated with liver attenuation.

This increased liver attenuation in cases of blood loss stands in opposition to both, the literature on the attenuation values of blood (mean 60 HU, range 40–90 HU) and liver parenchyma (weighted mean 50 HU, range mean 32–58 HU) on PMCT [12, 20] and the authors’ experience from liver attenuation measurements. Empirically, liver attenuation was often lower than the attenuation of blood in the portal (or hepatic) veins. This contradiction suggests the presence of a confounding factor.

There are several known causes for an increase in hepatic attenuation: According to Boll et al., the most frequent cause of elevated liver attenuation values on clinical non-contrast CT are high levels of hepatic glycogen [23]. Hepatic glycogen levels can quickly change in function of diet and the intake of a single glycogen-rich meal is sufficient to raise hepatic attenuation by nearly 5 HU, while depleted glycogen storage lowers the hepatic attenuation by about 5 HU from baseline in healthy male volunteers [24]. By contrast, other causes for increased liver attenuation such as iron or copper storage disease are not only rare, but they are chronic conditions, which evolve slowly over years [23, 25]. In view of this evidence, hepatic glycogen storage levels appear to be a likely candidate for the suspected confounding factor, if a difference between hepatic glycogen between cases and controls can be established.

Hepatic glycogen serves as stored fuel that can be released into the bloodstream as glucose if the body requires energy rapidly. Due to this, hepatic glycogen levels were historically used to estimate the duration of the agonal phase: High levels of hepatic glycogen were interpreted as a sign of a short agonal phase, with death occurring before the depletion of hepatic energy storages. Low levels of hepatic glycogen were attributed to a long/er agonal phase during which hepatic glycogen storages had been depleted. This method was introduced by the French physician and criminologist Lacassagne in the late nineteenth century and was later refined by the Austrian forensic pathologist Carl Meixner who confirmed the connection between high levels of hepatic glycogen and a short agonal phase [26, 27].

This connection between levels of glycogen in the liver and the duration of the agonal phase [26, 27] is relevant for the interpretation of the liver attenuation values in this study: It is possible that the high mean liver attenuation values in the case group were not caused by the loss of blood, but by the residual presence of glycogen. This interpretation would mean that the high liver attenuation values found in cases with significant hemorrhage were rather a marker of a short agonal phase than of the actual blood loss. Another possibility of the influence of agony time on liver attenuation could be the association of prolonged agony with multiorgan failure, which could influence liver attenuation. A strong indicator for the influence of agony on liver attenuation rather than blood loss is found in the subgroup analysis (Fig. 3). Two causes of death present with much lower attenuations: internal and iatrogenic hemorrhages. These are the two causes of death within the case group in which a higher average agony time can be strongly assumed.

Estimating agony times is notoriously difficult and literature on this topic is old and scarce. According to a classification system of agonal phases from 1965 [28], deaths related to hemorrhage tend to have shorter agony times than non-hemorrhage-related deaths. Based on this crude classification, it is reasonable to assume that the mean agony time of the case group with blood loss was shorter than the mean agony time of the control group without blood loss (see Fig. 2 for an overview of causes of death for cases and controls). Connecting this assumption with the findings of Lacassagne and Meixner [26, 27] further implies that hepatic glycogen levels also ought to have been higher in the case group than in the control group. Finally, the association between hepatic glycogen levels and hepatic attenuation on CT [21, 22] suggests that high liver attenuation values on CT in cases with hemorrhage is not a sign of blood loss, but a sign of a relatively short agony time—which is thought to be linked with hemorrhage related deaths [26]. Prospective studies with quantitative analysis of liver glycogen levels as well as case-by-case estimations of agony times are required to investigate the potential correlation between hepatic attenuation on CT and agonal phases.

The beta-coefficients of other variables included in the models for reasons of controlling for possible confounding (Age, Sex, BMI, PMI, liver and lung weight) factors presented with small effect sizes (Fig. 3)—although partially statistically significant. An exception is attempted CPR, which shows a substantial effect on both liver and lung attenuation. This is likely the result of intravenous volume administration during CPR attempts which increases the volume status but decreases the hematocrit, similarly described in cases 75 and 77 in Schober et al. [11]. The significance of the organ weights very likely describes no causal relationship between weight and attenuation. Instead, it is explained with the correlation of the organ weight to other covariates. As seen in the weight analysis, increased lung weights are strongly associated with the assignment to the control group and therefore capture some of the variance of the grouping variable (blood loss). The same is true for liver weight, but less pronounced, as the weight difference between the two groups is lower.

This study contains several limitations. It may be criticized that the attenuation of the examined organs was measured with ROI instead of full segmentation. To keep the methodology practical for routine application, ROI measurements were favored over organ segmentation. Organ segmentation is more time-consuming and requires dedicated software. Also, there is evidence in the literature that ROI measurements feature a high reproducibility within an absorber and have no significant intra- or interrater variability [16]. A second limitation of the study is that hepatic fat content was only evaluated macroscopically. The authors acknowledge this limitation. Hepatic fat content is not routinely quantified microscopically at the authors’ institution and due to the retrospective approach of this study, the information was no longer available. However, since this limitation affected both cases and controls, and the proportions of macroscopic liver fat were not significantly different, a selection bias is unlikely to have affected the difference in means of liver attenuation. For future investigations regarding the association of agony times, glycogen, hepatic steatosis, and blood loss with liver attenuation, a prospective microscopic evaluation of hepatic steatosis is strongly preferred.

## Conclusion

Blood loss decreases organ weight as well as CT attenuation of the lungs but appears to have no significant effect on CT attenuation of the spleen and kidneys. CT attenuation of the liver was higher in cases with blood loss than in controls without blood loss. The connection between hepatic attenuation on CT and hepatic glycogen levels as well as historic work on agony times suggests that high liver attenuation values on PMCT are rather an indicator of a relatively short agony time than a sign of blood loss. However, further dedicated work is required to investigate the potential impact of agony time on hepatic attenuation on PMCT.

## Declarations

This article does not contain any studies with live human participants or animals performed by any of the authors. Ethical approval for the publication of case-related data was obtained by the Ethics Committee of the Canton of Zurich, Nr. KEK ZH-Nr. 15–0686.

## References

[1] Feldschuh J, Enson Y (1977). Prediction of the normal blood volume. Relation of blood volume to body habitus. Circulation..

[2] Muldowney FP (1957). The relationship of total red cell mass to lean body mass in man. Clin Sci.

[3] Pacagnella RC, Souza JP, Durocher J, Perel P, Blum J, Winikoff B (2013). A systematic review of the relationship between blood loss and clinical signs. Plos one..

[4] Betz P (2004) Vitale Reaktionen und Zeitschätzungen. In: Brinkmann B, Madea B (eds) Handbuch gerichtliche Medizin. Springer-Verlag, Berlin, Heidelberg, pp 297–298

[5] Boyd E (1933). Normal variability in weight of the adult human liver and spleen. Arch Pathol Lab Med.

[6] Moar J, Reinach S (1988). Renal weights in the southern African black population. Am J Phys Anthropol.

[7] Myers J, Segal R (1974) Weight of the spleen. I. Range of normal in a nonhospital population. Arch Pathol 98(1):33–35 4829772

[8] Hadley JA, Fowler DR (2003). Organ weight effects of drowning and asphyxiation on the lungs, liver, brain, heart, kidneys, and spleen. Forensic Sci Int.

[9] Aghayev E, Sonnenschein M, Jackowski C, Thali M, Buck U, Yen K (2006). Postmortem radiology of fatal hemorrhage: measurements of cross-sectional areas of major blood vessels and volumes of aorta and spleen on MDCT and volumes of heart chambers on MRI. Am J Roentgenol.

[10] Sogawa N, Michiue T, Ishikawa T, Inamori-Kawamoto O, Oritani S, Maeda H (2015). Postmortem CT morphometry of great vessels with regard to the cause of death for investigating terminal circulatory status in forensic autopsy. Int J Legal Med.

[11] Schober D, Schwendener N, Zech W-D, Jackowski C (2017). Post-mortem CT: Hounsfield unit profiles obtained in the lungs with respect to the cause of death assessment. Int J Legal Med.

[12] Tisch C, Brencicova E, Schwendener N, Lombardo P, Jackowski C, Zech W-D (2019). Hounsfield unit values of liver pathologies in unenhanced post-mortem computed tomography. Int J Legal Med.

[13] Chatzaraki V, Verster J, Tappero C, Thali MJ, Schweitzer W, Ampanozi G (2019) Spleen measurements with reference to cause of death and spleen weight estimation: a study on postmortem computed tomography. J Forensic Radiol Imaging 18:24–31

[14] Huda W, Slone RM (2003) Review of radiologic physics: Lippincott Williams & Wilkins

[15] Jackowski C, Thali M, Aghayev E, Yen K, Sonnenschein M, Zwygart K (2006). Postmortem imaging of blood and its characteristics using MSCT and MRI. Int J Legal Med.

[16] Ruder TD, Thali Y, Schindera ST, Dalla Torre SA, Zech W-D, Thali MJ (2012). How reliable are Hounsfield-unit measurements in forensic radiology?. Forensic Sci Int.

[17] Pourhoseingholi MA, Baghestani AR, Vahedi M (2012). How to control confounding effects by statistical analysis. Gastroenterology and hepatology from bed to bench.

[18] Landis RS (2005) Standardized Regression Coefficients In: Balakrishnan N, Colton T, Everitt B, Piegorsch W, Ruggeri F, Teugels JL, editors. Wiley StatsRef: Statistics Reference Online2005.

[19] R Development Core Team (2019) A language and environment for statistical computing. Vienna, Austria: R Foundation for Statistical Computing

[20] Zech W-D, Jackowski C, Buetikofer Y, Kara L (2014). Characterization and differentiation of body fluids, putrefaction fluid, and blood using Hounsfield unit in postmortem CT. Int J Legal Med.

[21] Schweitzer W, Thali M, Giugni G, Winklhofer S (2014). Postmortem pulmonary CT in hypothermia. Forensic Sci Med Pathol.

[22] Schweitzer W, Thali M (2019). Fatal obstructive asphyxia: Trans-pulmonary density gradient characteristic as relevant identifier in postmortem CT. J Forensic Radiol Imaging.

[23] Boll DT, Merkle EM (2009). Diffuse liver disease: strategies for hepatic CT and MR imaging. Radiographics.

[24] Leander P, Sjöberg S, Höglund P (2000). CT and MR imaging of the liver: clinical importance of nutritional status. Acta Radiol.

[25] Ashraf A, Weerakkody Y. Hepatic Attenuation on CT [Available from: https://radiopaedia.org/articles/hepatic-attenuation-on-ct?lang=us.

[26] Lacassagne A, Martin É (1899). De la docimasie hépatique. Les Archives d'anthropologie criminelle.

[27] Meixner C (1911). Das Glykogen der Leber bei verschiedenen Todesarten. Beitr Gerichtl Med.

[28] Laves W, Berg S. Agonie: Physiologisch-chemische Untersuchungen bei gewaltsamen Todesarten: Schmidt-Römhild; 1965.

